# Use of Corn Silk Meal in Broiler Diet: Effect on Growth Performance, Blood Biochemistry, Immunological Responses, and Growth-Related Gene Expression

**DOI:** 10.3390/ani11041170

**Published:** 2021-04-19

**Authors:** Abeer A. Kirrella, Safaa E. Abdo, Karima El-Naggar, Mohamed Mohamed Soliman, Salama Mostafa Aboelenin, Mahmoud A. O. Dawood, Ahmed A. Saleh

**Affiliations:** 1 Department of Poultry Production, Faculty of Agriculture, Kafrelsheikh University, Kafr El-Sheikh City 33516, Egypt; abeerabdelatty@yahoo.com; 2 Department of Animal Wealth Development, Faculty of Veterinary Medicine, Kafrelsheikh University, Kafr El-Sheikh City 33516, Egypt; safaa_2m@yahoo.com; 3 Department of Nutrition and Veterinary Clinical Nutrition, Faculty of Veterinary Medicine, Alexandria University, Alexandrina City 21500, Egypt; karima.muhammad@alexu.edu.eg; 4 Clinical Laboratory Sciences Department, Turabah University College, Taif University, P.O. Box 11099, Taif 21944, Saudi Arabia; mmsoliman@tu.edu.sa; 5 Biology Department, Turabah University College, Taif University, P.O. Box 11099, Taif 21944, Saudi Arabia; s.aboelenin@tu.edu.sa; 6 Department of Animal Production, Faculty of Agriculture, Kafrelsheikh University, Kafr El-Sheikh City 33516, Egypt; Mahmoud.dawood@agr.kfs.edu.eg

**Keywords:** corn silk, enzyme, broiler, growth performance, immune status

## Abstract

**Simple Summary:**

Corn silk meal (CSM), the outer thread-like material found under corn husks, is a by-product of corn cultivation, which has been documented as a traditional medicine with multiple benefits for human health. Broilers cannot produce the enzymes needed to hydrolyze non-starch polysaccharide (NSP), which has been found to increase gut viscosity, and reduce the digestion and utilization of nutrients, thereby resulting in poor animal performance. The objective of this study was to examine the effects of diets supplemented with CSM and NSP on growth performance, blood biochemistry, immunological and growth-related gene expression in broiler chickens. A total of 270 broiler chickens were divided into six experimental groups: (1) basal diets (BD) as control; (2) BD supplemented with 0.5 g/kg feed NSP enzyme; (3) and (4) fed a diet containing 40 and 80 kg/ton of CSM; (5) and (6) fed a diet containing 40 and 80 kg/ton CSM and supplemented with 0.5 g/kg NSP enzyme. The results showed that the dietary inclusion of CSM with the NSP enzyme can improve growth performance, intestinal histopathology parameters, modify plasma lipids, and enhance immune response in broilers.

**Abstract:**

The objective of this study was to examine the effects of diets supplemented with corn silk meal (CSM) and non-starch polysaccharide (NSP) enzyme on growth performance, blood biochemistry, immunological response, and growth-related gene expression in broiler chickens. A total of 270 broiler chickens were divided into six experimental groups: (1) basal diets (BD) as control; (2) BD supplemented with 0.5 g/kg feed NSP enzyme; the other four groups are CSM diets as following; (3) and (4) fed diet contain 40 and 80 kg/ton of CSM; (5) and (6) fed diet contain 40 and 80 kg/ton CSM and supplemented with 0.5 g/kg NSP enzyme. Body weight gain (BWG), feed conversion ratio (FCR), protein retention and fiber digestibility were synergistically improved (*p* < 0.05) when fed CSM supplemented with NSP enzyme. Moreover, a synergistic decrease (*p* < 0.05) in the serum glucose and total cholesterol were found. Immune organ weights and Newcastle disease virus titers were increased with CSM diets. Interestingly, the relative mRNAs of the growth hormone receptor (GHR) and insulin growth factor (IGF) were increased (*p* < 0.05) with the CSM and NSP enzyme: the relative mRNA expressions of cholecystokinin (CCK) and leptin were decreased by feeding CSM diets with the NSP enzyme. It could be concluded that the dietary inclusion of CSM with the NSP enzyme might improve growth performance, modify plasma lipids, and enhance immune response in broilers.

## 1. Introduction

Poultry feed is one of the highest expenses for poultry producers, and can constitute 60–70% of the total cost of broiler production [1]. Nutritionists have started searching for more economic alternatives to the expensive, traditional feedstuffs used in poultry diets, with the goal of reducing costs without compromising bird performance. Agro-industrial by-products are one such unconventional feed ingredient that could help decrease feed costs while increasing profits [1,2,3]; however, most of these products are high in fiber, low in protein, and contain other anti-nutritional factors that negatively affect digestibility.

Corn grains have several valuable by-products, such as corn silk (CS; Maydis stigma). The corn silk meal (CSM) exists as thready matter from under the husk of corn grains and is validated in phytotherapy to improve human health [4]. The functionality of CSM is attributed to its content of minerals (Na, Ca, K, Fe, Ze, Cl) [5], vitamins (E and K), flavonoids, phytosterols, volatiles oils, steroids, saponin, alkaloids, tannins, and allantoin [6,7]. Previous studies have reported that CS has the potential to be used as an antioxidant and in health care [8,9,10] for its antibacterial [11,12] anti-inflammatory, antiviral, anti-allergic [13], anti-hyperlipidemic [14], and anti-diabetic [15] properties. In this regard, the dietary inclusion of CSM as an alternative feed ingredient in poultry diets could help reduce production costs, promote health and improve broiler performance; however, a primary concern about its incorporation into poultry diets is its high total fiber content (CF, 9.5%), which has been shown to have a detrimental impact on nutrient utilization efficiency and consequently, broiler performance.

Usually, broiler’s feed is formulated by including traditional ingredients, such as corn and soybean meal. These ingredients’ cell walls have a high level of polysaccharides at 29% in soybeans and 9% in corn [16]. Broilers cannot produce the enzymes needed to hydrolyze non-starch polysaccharides (NSPs) [17,18], which have been found to increase gut viscosity and reduce the digestion and utilization of nutrients, thereby resulting in poor animal performance [19]. Supplemental exogenous enzymes have been reported to overcome the problem of using CSM by increasing fibrous material digestion, absorption and nutrient utilization, thereby enhancing gut immunity, improving health, and increasing broiler performance by [20].

In the present study, we intended to investigate the beneficial effects of supplementing CSM and/or NSP enzymes in broiler diets. We gathered data on growth performance, blood biochemistry, carcass traits, immunological, and histological responses, as well as growth-related and feed intake-related gene expression in broilers.

## 2. Materials and Methods

### 2.1. Experimental Design

This study was approved by the Ethics Committee of Local Experimental Animals Care Committee and was conducted in accordance with the guidelines of Kafrelsheikh University, Egypt (Number 4/2016 EC). A total of 300 1-day-old, male broiler chicks (Cobb Avian 48) were housed in an electrically heated brooder and given free access to water and a commercial starter diet (23% crude protein and metabolizable energy (ME) of 3000 kcal/kg until day 7 of age. On day 7, 270 chicks with a similar body weight were selected from the 300 chicks and were housed in pens. The chicks were divided into six treatments, each treatment was divided into five replicates and each replicate had 9 birds the groups as following; (1) birds fed the basal diet (BD) as control; (2) birds fed the BD supplemented with 0.5 g/kg feed NSP enzyme; (3) and (4) birds fed the experimental diets containing 40 and 80 kg/ton CSM; (5) and (6) birds fed the same diet as group 3 and 4, but with the addition of 0.5 g/kg NSP enzyme. All the birds were fed the experimental diets (grower and finisher) from day 7 to 35. All experimental diets were formulated to meet the nutrient requirements for poultry [21]. The composition and chemical analysis of the different experimental diets are presented in Table 1. Birds had free access to feed and water for the entire five-week experimental period. Matured corn silk was obtained from the Kafrelsheikh University farm after summer season. The fresh corn silk was shade- dried for 5 days. The dried corn silks samples were ground into powder using a grinder then kept in plastic bags at 4 °C until used. The chemical composition of corn silk meal is presented in Table 2. The enzyme formulations used were: Hemicell1 (0.033% *w*/*w*; CLH diet; endo-1,4-β-D-mannanase above 16 × 10^4^ U/g, Elanco Company, Greenfield, IN, USA). The feed trial took place in a temperature-controlled room with an 18 h light and 6 h dark cycle, at a temperature of starting from 29 ± 1 and decreasing by 1 °C every three days until 24 ± 1 °C and kept until end of the experiment (35 days), and proportional humidity between 50% and 70%. Throughout the experimental phase the health status and mortalities were measured daily on a regular basis.

### 2.2. Sampling

Chicken body weight (BW) and feed intake (FI) were recorded every week. Body weight gain (BWG) and feed conversion ratio (FCR) were calculated from the recorded data. Two birds from each replicate (10 birds/treatment) at the end of the experiment (35 days of age) were arbitrarily chosen and euthanized, then the blood samples were collected in clean vials for serum separation. Serum was stored at −20 °C for further analysis. Liver tissues were collected for gene expression. The carcass weight and different organ weights (pectoral superficial muscle, thigh muscle, thymus, bursa of Fabricius, spleen, liver, and abdominal fat) were recorded.

### 2.3. Serum Biochemical Analysis

Total protein, albumin, total cholesterol, high and low-density lipoproteins (HDL and LDL), alanine aminotransferase (ALT), aspartate aminotransferase (AST), glucose, creatinine, and triiodothyronine (T3) were measured colorimetrically using commercial kits (Diamond Diagnostics, Cairo, Egypt) according to the procedure outlined by the manufacturer [22].

### 2.4. Serum Antibody Titers

An HI test was used to examine serum samples for the presence of hemagglutination inhibiting antibodies to the Newcastle Disease Virus (ND) and avian influenza (H9N1). Two-fold serial dilutions of the test samples were mixed with an equal volume of NDV antigen. Chicken red blood cells (CRBCs) were added and subsequently the dilutions were examined for the presence of the complete inhibition of the hemagglutination—according to the OIE [23].

### 2.5. Nutrient Digestibility

For the final three experiment days, birds (*n* = 10 birds per treatment at same body weight) were housed individually in special metabolic cages (40 × 40 × 50 cm) in order to determine nutrient digestibility. During this period, birds and FI were weighed daily. Excreted feces were also collected, weighed daily, then stored in a freezer. After the digestibility experimental period, all samples were dried in a drying oven at 60 °C for 24 h. The whole dried samples were then homogenized. Samples were used for analysis according to procedures given by the Association of Official Analytical Chemists [24]. The crude protein concentrations in the diet and excreta were measured to determine nitrogen retention using the Kjeldahl method (AOAC 945.38 F), while crude fibers analysis method (CF, Method 932.09). The calculation was as follows: nitrogen retention (%) = (total nitrogen intake − total nitrogen excreted)/total nitrogen intake × 100 [25,26].

### 2.6. RNA Extraction and Real-Time PCR

#### 2.6.1. RNA Isolation and cDNA Synthesis

Liver tissue samples (*n* = 10) were collected and placed in liquid nitrogen, then stored at −80 °C until analysis. The total RNA was extracted using TRI reagent (easy-RED™, iNtRON Biotechnology, South Korea), following the manufacturer’s protocol. The integrity and quality of RNA was verified using 2% agarose gel electrophoresis. The RNA concentration was determined using Nano drop (UV–Vis spectrophotometer Q5000, Quawell, San Jose, CA, USA). The RNA sample was reverse transcribed to the first strand cDNA using the SensiFAST™ cDNA synthesis kit (Bioline, UK), then stored at −20 °C until analysis.

#### 2.6.2. Q-PCR (Quantitative Real-Time Polymerase Chain Reaction)

Gene-specific primer sequences (Table 3) were used to amplify the selected genes. The primer sequences were verified using UCSC In silico PCR (https://genome.ucsc.edu/cgi-bin/hgPcr, 10 March 2018) and NCBI primer blast. Real-time was performed using the Stratagene MX300P Realtime PCR machine (Agilent Technologies, CA, USA), with the SensiFast™ SYBR Lo-Rox kit (Bioline, UK) following the manufacturer’s recommendations. The amplification protocol was: initial denaturation at 95 °C for 15 min, followed by 40 cycles at 95 °C for 15 s, and an annealing temperature (specific for each primer, Table 2) for 1 min [27,28,29]. Dissociation curves were analyzed in order to verify that only one peak was found for each specific melting temperature, thus showing that the PCR products were amplified specifically. All samples were tested in duplicates. Target gene Ct values were first normalized against the Ct values of the house-keeping gene (β actin), then used to calculate the relative gene expression levels (fold change) according to [30]. RNA extraction, cDNA synthesis and real-time PCR were completed at the plant pathology and Biotechnology laboratory and EPCRS Excellence Center, Faculty of Agriculture, Kafrelsheikh University, Egypt.

### 2.7. Statistical Analysis

The differences between treatments were statistically analyzed using the two-way analysis of variance test in a completely randomized design with SPSS Statistics 17.0 (Statistical Packages for the Social Sciences, released 23 August 2008). Tukey’s multiple comparison test was used to identify which treatment conditions were significantly different from each other at a significance level of *p* < 0.05. Two-way ANOVA was applied to determine the main effects of corn silk meal or NSP enzyme and an interaction between them, when pens were the statistical units for performance parameters, birds for carcass, organs weights and samples for biochemical and other parameters.

## 3. Results

### 3.1. Growth Performance and Organs Weight

The effects of feeding CSM and/or NSP enzyme supplementation on broiler growth performance and nutrient utilization are presented in Table 4. Growth performance (body weight, feed intake, body weight gain and feed conversion ratio) were not affected by experimental groups until 14 days, except FCR which improved by NSP enzyme and 4% CSM with enzymes compared with other groups.

At 21 days, the body weight and body weight gain were increased significantly by feeding control diet with enzyme and 4% CSM with enzyme on the other hand, feed intake was not affected. After 28 days, the body weight body and weight gain were increased and FCR was improved by enzyme and 4% CSM with enzyme but feed intake decreased only by an enzyme group. Finally, the birds fed on a diet containing 4% of CSM and supplemented with enzyme showed the highest final body weight when compared with the control or those fed the same diet without enzyme supplementation. The body weight gain followed the same trend observed with BWG. Feed intake was not affected between the different experimental groups. The effect of our treatments on BWG and FI affected the FCR, which was obviously improved in the birds which received a diet containing 4% CSM and supplemented with enzymes. Crude protein retention was significantly improved by feeding CSM with enzyme; however, crude fiber digestibility was improved by supplementing enzyme in the basal diets and 4% CSM with enzyme. Throughout the experimental phase, no mortalities were recorded in the experiment period.

As shown in Table 5, birds’ carcass, breast muscle, thigh muscle, bursa of fabricius and thymus weights were increased (*p* < 0.05 and *p* < 0.01 for breast muscle) with the dietary inclusion of CSM and the mixture of CSM and enzyme compared with their reference groups. No differences were detected in liver weight and abdominal fat. However, spleen weight increased significantly by CSM with the enzyme and control group compared with other groups.

### 3.2. Blood Biochemical Traits

Data for the serum biochemical constituents are shown in Table 6. The level of total cholesterol was reduced (*p* < 0.05) with CSM containing a diet separately and in combination with enzyme addition. However, the total protein, albumin, ALT, AST, LDL and antibody titer of influenza were not influenced. On the other hand, HDL and the antibody titer of Newcastle were increased (*p* < 0.05) in birds fed CSM and/or the NSP enzyme. A significant interaction between the factors of enzymes and CSM were observed in serum glucose or in CSM 4% with enzyme supplementation, which significantly decreased serum glucose compared with the control and 4% CSM groups, while the birds fed CSM and NSP enzyme separately resulted in a significant increase in concentrations of triiodothyronine (T3).

### 3.3. Gene Expression

**Figure 1:**
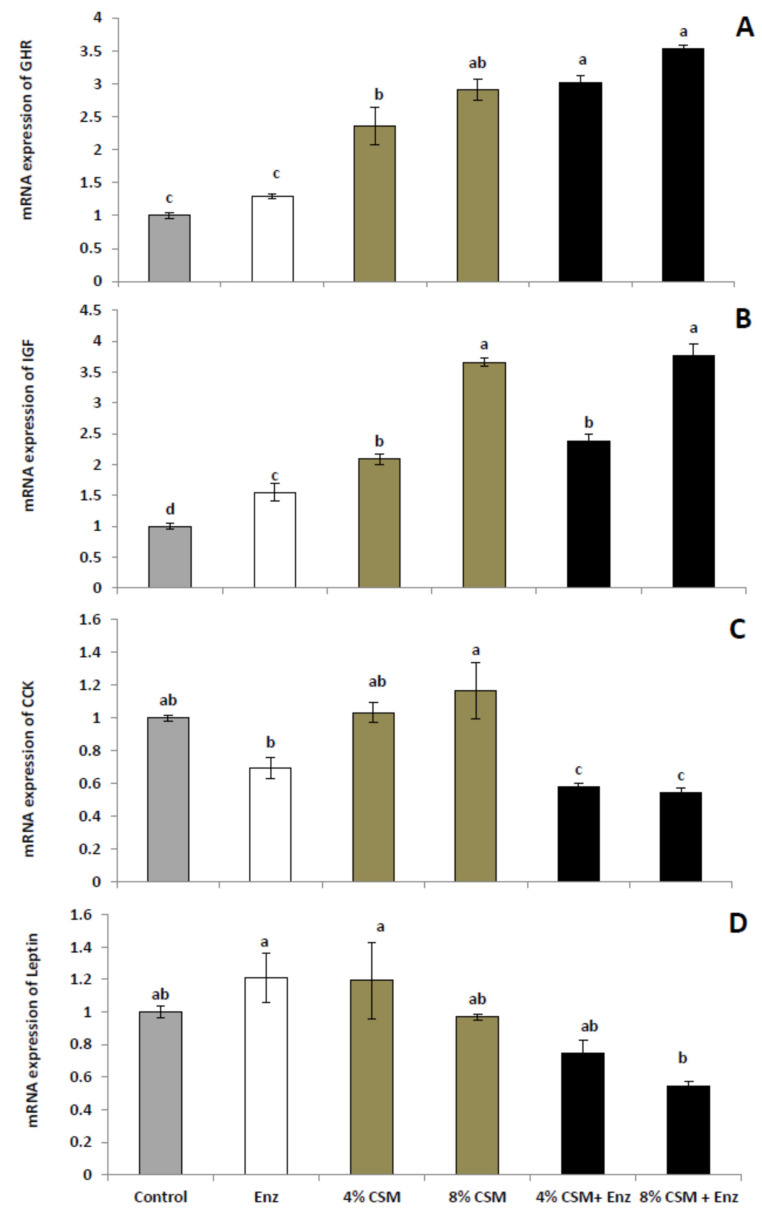
Effects of feeding corn silk meal and/or NSP enzyme mRNA of genes-related growth (Growth Hormone Receptor (GHR) (**A**), Insulin Like Growth Factor (IGF), (**B**) and feed intake regulatory genes (Cholecystokinin (CCK) (**C**) and Leptin (**D**).

The mRNA expression of growth regulatory genes, GHR and IGF were up-regulated by CSM dietary inclusion at 4% and 8% (*p* < 0.05) (Figure 1). The degree of up regulation was higher in birds fed CSM containing a diet with the addition of enzyme (CSM with NSP Enzyme) (*p* < 0.001). Moreover, the up-regulated gene expression of IGF showed a dose-dependent effect. Relative feed intake inhibitory gene, CCK gene expressions was modified by 4% and 8% CSM with enzyme but 8% CSM with enzyme significantly decreased gene expression of leptin compared to the enzyme alone and 4% CSM group (Figure 1). Regardless of the CSM level included in diet, CSM dietary inclusion with the NSP enzyme significantly lowered the expression of CCK (*p* < 0.001) compared with others groups; on the other hand, the highest level of the IGF gene was obtained by 8% CSM with or without the enzyme.

## 4. Discussion

This study presented an enhanced body weight gain (BWG) and feed conversion ratio (FCR) in birds delivered CSM and/or NSP enzyme, which can be attributed to the enhanced digestibility of CSM due to the effect of an NSP enzyme. In addition, the high nutritional value of CSM (crude protein and metabolizable energy) resulted in improved intestinal morphometrical features leading to the efficient digestion and absorption of nutrients, thereby growth performance and antioxidative capacity [31,32]. The results are similar with relevant investigations which reported the potential role of CSM in maintaining the health and wellbeing of different mammalian and avian species [33,34].

The improved FCR reflects the role of NSP in regulating the digestibility of feed and the availability of digested nutrients for metabolic functions [35]. The activation of the NSP enzyme is also associated with its role in enhancing the diversity of beneficial microbiota in birds’ gut [36]. These results may explain the enhanced feed digestion and growth performances in broilers fed CSM and the NSP enzyme. Similarly, Slominski et al. [37] stated that using a mixture of digestive enzymes led to enhanced nutrient digestibility and the elimination of negative impacts of NSPs. Furthermore, Ravn et al. [38] added that the mixture of enzymes resulting in improved villi length in the bird’s gut led to high feed utilization and growth performance. This study also presented improved nitrogen retention and ME in birds fed CSM and the NSP enzyme, which correlated with improved feed digestibility.

Feeding CSM and/or the NSP enzyme resulted in the high weights of muscles, bursa, thymus, and spleen organs which were probably attenuated by improved protein synthesis. This stimulated increase in organ weights was shown to be dependent on the level of CSM inclusion in diet. It has been reported that including an NSP enzyme in broilers feed resulted in high breast muscle weights, protein synthesis, and upregulated insulin-like growth factor receptor (IGFR) [39,40]. The increased weight of immune organs (bursa, thymus, and spleen) is usually associated with the enhanced immune function and health conditions of birds [41]. The inclusion of CSM in broilers feed attenuated the immunity and led to the high weight of immune organs [42].

This study showed increased blood total cholesterol, and reduced HDL in birds fed CSM. In parallel, the extracts of corn silk caused low cholesterol, triglycerides, and LDL in mice [43] and rats [14]. Serum, and total cholesterol were reduced by the experimental diets, while HDL was increased. Similarly, lower levels of total cholesterol, triglycerides, and LDL were detected in rats [14] and hyperlipidemic mice [43] when total flavonoids from corn silk extract were added to their diets. In addition, Zhang et al. [44] concluded that the levels of TC, TG, LDL, and HDL were decreased in mice treated with CSM-derived flavonoids. The authors attributed the regulated lipids levels in the blood to the absence of oxidative stress and the high ability of CSM as an antioxidant agent involved in lowering lipid peroxidation.

The results showed low glucose levels in birds treated with the CSM and NSP enzyme, indicating non-stressful conditions while an increased triiodothyronine (T3) level, which agrees with [15,45]. The ND antibody titers showed enhanced values in birds treated with CSM with or without NSP enzyme, but no marked effect was seen on avian influenza (H9N1). Concurrent with the increased weight of bursa Fabricus, which refers to enhanced immunity and resistance to pathogenic infection resulting from CSM feeding. Indeed, NSP has abundant amounts of polysaccharides which may result in activated immunity [46]. Catap et al. [47] reported marked enhancements in the immunity and resistance against the infection of fish delivered CSM-treated feeds. The authors attributed enhanced immunity to the high content of CSM from polysaccharide, monosaccharide, flavonoid, and alkaloid derivatives. Consistently with this study, the references [39,40] concluded that there were enhanced ND and infectious bronchitis disease antibody titers in broilers treated with NSP enzymes.

The growth behavior of birds is the final response of feed utilization and wellbeing resulting from the high metabolic rate of nutrients and deposition of proteins which can be influenced by the chemical composition of diets and ingredients [48]. Furthermore, the deposition of nutrients, muscle tissue development [49], and feed utilization can be regulated by the IGF-1 gene [50], which is released under the control of GH and GHR [51]. Additionally, the feed content can affect the level of consumed feed and thereby the growth rate probably by affecting the balance of relevant genes (e.g., CCK) [52]. The results showed upregulated GHR and IGF-1 genes but downregulated CCK and leptin genes in birds fed CSM and NSP enzyme. These results agree with [53], who showed an upregulated sodium–glucose cotransporter 1 and IGFR genes in broilers fed dietary NSP enzymes. Furthermore, [54] reported that the inclusion of the NSP enzyme improved the expression of GHR and IGFR genes. In addition, CSM content high polyphenols which have good roles in enhancing the gene expressions related to growth [55]. Although FI was not affected by the treatments, the FI inhibitory genes, leptin, and CCK were downregulated by CSM and NSP enzyme supplementation.

## 5. Conclusions

We conclude that feeding CSM with endo-1,4-β-D-mannanase had positive effects on growth performance, nutrient digestibility, immune response, blood biochemistry, and growth-related gene expression in broiler chickens.

## Figures and Tables

**Table 1:** Composition of the experimental diets.

	Control		Enzyme		4% CSM		8% CSM		4% CSM + Enz		8% CSM + Enz	
Ingredients (g/kg)	Grower	Finisher	Grower	Finisher	Grower	Finisher	Grower	Finisher	Grower	Finisher	Grower	Finisher
Yellow corn	577	637	576.5	636.5	550	609	508	569	549.5	608.5	507.5	568.5
Soybean meal (46%)	328	260	328	260	322	256	320	248	322	256	320	248
Gluten (62%)	15	25	15	25	18	26	17	30	18	26	17	30
Soybean oil	30	29	30	29	30	30	35	34	30	30	35	34
Dicalcium phosphate	13	15	13	15	13	15	13	15	13	15	13	15
DL-Methionine	3.2	2.6	3.2	2.6	3.2	2.6	3.2	2.6	3.2	2.6	3.2	2.6
L-Lysine	2	3	2	3	2	3	2	3	2	3	2	3
Threonine	0.8	0.9	0.8	0.9	0.8	0.9	0.8	0.9	0.8	0.9	0.8	0.9
CaCo_3_	13	9	13	9	13	9	13	9	13	9	13	9
NaCl	3.5	3.5	3.5	3.5	3.5	3.5	3.5	3.5	3.5	3.5	3.5	3.5
Premix *	3	3	3	3	3	3	3	3	3	3	3	3
NaHCO_3_	1.5	2	1.5	2	1.5	2	1.5	2	1.5	2	1.5	2
CSM **					40	40	80	80	40	40	80	80
NSP enzyme			0.5	0.5					0.5	0.5	0.5	0.5
Calculated analysis (%)												
Crude Protein	21.04	19.06	21.04	19.06	21.06	19.05	21.05	19.05	21.06	19.04	21.04	19.04
ME (Kcal/kg)	3044	3116	3043	3115	3042	3114	3045	3115	3044	3113	3043	3115
Calcium	0.892	0.766	0.892	0.766	0.901	0.775	0.902	0.765	0.901	0.765	0.902	0.765
Av. P	0.493	0.491	0.493	0.491	0.493	0.491	0.496	0.493	0.492	0.491	0.496	0.492
Crude Fiber	3.304	3.081	3.303	3.080	3.484	3.266	3.754	3.518	3.483	3.265	3.753	3.516
Na	0.204	0.216	0.204	0.216	0.207	0.219	0.201	0.213	0.207	0.219	0.201	0.213
Cl	0.251	0.251	0.251	0.251	0.254	0.253	0.257	0.256	0.254	0.253	0.257	0.256
Digestible Lysine	1.306	1.199	1.306	1.198	1.303	1.196	1.308	1.191	1.304	1.196	1.307	1.191
Digestible Methionine	0.676	0.601	0.676	0.600	0.669	0.592	0.658	0.585	0.669	0.591	0.657	0.585
Digestible Threonine	0.853	0.758	0.852	0.758	0.844	0.751	0.842	0.745	0.844	0.748	0.842	0.745
Chemical analysis (%)												
Crude Protein	21.10	19.02	21.11	19.02	21.09	19.04	21.05	19.03	21.08	19.04	21.07	19.05
Crude Fiber	3.297	3.051	3.298	3.050	3.348	3.195	3.632	3.498	3.351	3.212	3.650	3.489
Ether extract	4.461	5.65	4.461	5.652	4.453	5.643	4.455	5.652	4.460	5.621	4.462	5.648

* Premix (Hero mix^®^, Hero pharm, Cairo, Egypt). Each 3 kg vitamin and mineral mixture contain: vitamin A 12,000,000 IU; vitamin D3 2,500,000 IU; vitamin E 10,000 mg; vitamin K3 2000 mg; vitamin B1 1000 mg; vitamin B2 5000 mg; vitamin B6 1500 mg; vitamin B12 10 mg; niacin 30,000 mg; biotin 50 mg; folic acid 1000 mg; pantothenic acid 10,000 mg; manganese 60,000 mg; zinc 50,000 mg; iron 30,000 mg; copper 4000 mg; iodine 300 mg; selenium 100 mg; and cobalt 100 mg). ** CSM: corn silk meal.

**Table 2:** The chemical composition of the corn silk meal.

Dry matter	95.9%
Crude protein	12.51%
Crude fiber	9.5%
Metabolizable energy (ME)	2550 Kcal/Kg
Calcium	0.31%
Total phosphorus	0.32%
Available phosphorus	0.24%
Digestible lysine	0.62%
Digestible methionine	0.23%
Digestible threonine	0.36%

**Table 3:** Gene-specific primers sequence used in the experiment.

Gene	Accession No.	Primer	Ref.	Annealing
β-actin	NM_205518.1	F: ACCTGAGCGCAAGTACTCTGTCT R: CATCGTACTCCTGCTTGCTGAT	[25]	60
Growth Hormone Receptor (GHR)	NM_001001293.1	F: AACACAGATACCCAACAGCC R: AGAAGTCAGTGTTTGTCAGGG	[27]	60
Insulin Like Growth Factor (IGF)	NM_001004384.2	F: CACCTAAATCTGCACGCT R: CTTGTGGATGGCATGATCT	[27]	60
Leptin	AF082500	F: TCCGCCAAGCAGAGGGGT R: CCAGGACGCCATCCAGGCTCTCTGGC	[28]	58
Cholecystokinin (CCK)	NM_001001741.1	F: CAGCAGAGCCTGACAGAACC R: AGAGAACCTCCCAGTGGAACC	[29]	58

**Table 4:** Effects of feeding corn silk meal and/or non-starch polysaccharide (NSP) enzyme on the growth performance and nutrient digestibility.

	Treatments						ANOVA		
	Control	Enz.	4% CSM	8% CSM	4% CSM + Enz.	8% CSM + Enz	Enz	CSM	Enz × CSM
Initial body weight, g at 7 d	152.3 ± 0.1	152.3 ± 0.03	152.1 ± 0.16	152.1 ± 0.01	152.3 ± 0.02	152.3 ± 0.02	NS	NS	NS
Feed intake, g 0–7 d	116.2 ± 0.41	116.4 ± 0.34	116.2 ± 0.21	116.4 ± 0.32	115.8 ± 0.32	115.7 ± 0.36	NS	NS	NS
FCR, 7 d	0.763 ± 0.007	0.764 ± 0.002	0.764 ± 0.004	0.765 ± 0.003	0.760 ± 0.004	0.760 ± 0.005	NS	NS	NS
Body weight, g at 14 d	456 ± 4	462 ± 5	455 ± 5	460 ± 6	468 ± 8	461 ± 7	NS	NS	NS
Body weight gain, g (7–14) d	304 ± 6	310 ± 5	303 ± 5	308 ± 6	316 ± 7	309 ± 7	NS	NS	NS
Feed intake, g 7–14 d	462 ± 5	466 ± 3	461 ± 7	469 ± 4	471 ± 7	468 ± 5	NS	NS	NS
FCR, 14d	1.519 ± 0.006 ^ab^	1.503 ± 0.008 ^b^	1.522 ± 0.007 ^a^	1.523 ± 0.009 ^a^	1.491 ± 0.008 ^b^	1.515 ± 0.006 ^ab^	*	*	NS
Body weight, g at 21 d	932 ± 12 ^b^	954 ± 15 ^a^	930 ± 17 ^b^	941 ± 16 ^ab^	966 ± 15 ^a^	944 ± 14 ^ab^	*	*	NS
Body weight gain, g (14–21) d	476 ± 8 ^b^	492 ± 8 ^a^	475 ± 7 ^b^	481 ± 8 ^ab^	498 ± 9 ^a^	483 ± 6 ^ab^	*	*	NS
Feed intake, g 14-21 d	716 ± 23	704 ± 21	712 ± 19	710 ± 18	701 ± 21	706 ± 22	NS	NS	NS
FCR, 21d	1.504 ± 0.007 ^a^	1.431 ± 0.006 ^bc^	1.499 ± 0.007 ^ab^	1.476 ± 0.009 ^ab^	1.408 ± 0.004 ^c^	1.462 ± 0.008 ^ab^	*	NS	*
Body weight, g at 28 d	1502 ± 25 ^b^	1553 ± 29 ^a^	1506 ± 37 ^b^	1523 ± 16 ^ab^	1654 ± 42 ^a^	1538 ± 36 ^ab^	*	*	NS
Body weight gain, g (21–28) d	570 ± 12 ^c^	599 ± 14 ^b^	576 ± 13 ^c^	582 ± 15 ^bc^	688 ± 17 ^a^	594 ± 16 ^b^	*	*	*
Feed intake, g 21–28d	1010 ± 37	998 ± 39	1003 ± 28	1005 ± 32	1009 ± 34	1008 ± 38	NS	NS	NS
FCR, 28d	1.772 ± 0.01 ^a^	1.650 ± 0.05 ^bc^	1.741 ± 0.07 ^ab^	1.727 ± 0.05 ^ab^	1.467 ± 0.08 ^c^	1.697 ± 0.07 ^ab^	*	NS	*
Body weight, g at 35 d	1802 ± 51 ^b^	1854 ± 28 ^ab^	1796 ± 54 ^b^	1832 ± 28 ^ab^	1942 ± 23 ^a^	1832 ± 32 ^ab^	NS	NS	*
Body weight gain, g (28–35) d	300 ± 12 ^ab^	301 ± 17 ^a^	290 ± 13 ^b^	309 ± 19 ^a^	280 ± 12 ^b^	294 ± 17 ^ab^	NS	*	*
Feed intake, g (28–35) d	1067 ± 46 ^a^	1015 ± 38 ^b^	1055 ± 43 ^a^	1062 ± 30 ^a^	1045 ± 34 ^ab^	1020 ± 31 ^b^	NS	NS	NS
Body weight gain, g (7–35) d	1650 ± 51.2 ^b^	1702 ± 28.1 ^ab^	1643 ± 53.8 ^b^	1680 ± 28.1 ^ab^	1790 ± 22.6 ^a^	1680 ± 31.5 ^ab^	NS	NS	*
Feed intake, g (7–35) d	3371 ± 46	3335 ± 38	3383 ± 43	3398 ± 30	3378 ± 34	3354 ± 31	NS	NS	NS
FCR, 7–35	2.043 ± 0.21 ^a^	1.959 ± 0.26 ^ab^	2.059 ± 0.23 ^a^	2.023 ± 0.31 ^a^	1.887 ± 0.18 ^b^	1.997 ± 0.20 ^ab^	NS	NS	*
Crude protein retention%	67.1 ± 0.37 ^c^	70.67 ± 0.41 ^b^	67.57 ± 0.23 ^c^	70.37 ± 0.37 ^b^	72.63 ± 0.29 ^a^	72.3 ± 0.26 ^a^	*	*	**
Crude fiber digestibility%	42.97 ± 0.203 ^b^	48.13 ± 0.745 ^a^	43.3 ± 0.208 ^b^	42.47 ± 0.59 ^b^	47.67 ± 1.38 ^a^	45.47 ± 1.41 ^ab^	*	*	*

Enz: enzyme; CSM: corn silk meal; FCR: feed conversion ratio. Values are expressed as the means ± standard error. Means within a row not sharing a common superscript significantly differ from each other. NS, not significant (*p* > 0.05); * *p* < 0.05; ** *p* < 0.01. Pens used as sample units.

**Table 5:** Effects of feeding corn silk meal and/or NSP enzyme on carcass, organs weights and abdominal fat weights.

	Treatments						ANOVA		
	Control	Enz	4% CSM	8% CSM	4% CSM + Enz	8% CSM + Enz	Enz	CSM	Enz × CSM
Carcass, g/100 g BW	59.87 ± 2.4 ^b^	66.61 ± 2.01 ^a^	65.8 ± 1.08 ^a^	66.92 ± 1.13 ^a^	65.71 ± 2.32 ^a^	64.85 ± 1.59 ^ab^	*	*	*
Breast muscle, g/100 g BW	21.26 ± 1.21 ^cd^	20.88 ± 0.63 ^d^	21.65 ± 0.36 ^bcd^	25.63 ± 1.08 ^a^	24.46 ± 1.18 ^ab^	23.99 ± 0.86 ^abc^	NS	*	**
Thigh muscle, g/100 g BW	12.59 ± 0.34 ^b^	13.97 ± 0.66 ^ab^	14.91 ± 0.87 ^ab^	15.02 ± 1.21 ^ab^	15.63 ± 1.48 ^ab^	17.18 ± 1.41 ^a^	NS	*	**
Bursa, g/100 g BW	0.093 ± 0.017 ^b^	0.13 ± 0.015 ^ab^	0.137 ± 0.016 ^ab^	0.147 ± 0.019 ^a^	0.146 ± 0.011 ^a^	0.141 ± 0.018 ^ab^	NS	*	*
Thymus, g/100 g BW	0.147 ± 0.014 ^c^	0.201 ± 0.019 ^bc^	0.244 ± 0.019 ^abc^	0.314 ± 0.033 ^a^	0.29 ± 0.047 ^ab^	0.284 ± 0.054 ^ab^	NS	*	**
Spleen, g/100 g BW	0.142 ± 0.016 ^a^	0.086 ± 0.018 ^b^	0.106 ± 0.008 ^ab^	0.122 ± 0.016 ^ab^	0.127 ± 0.006 ^a^	0.129 ± 0.005 ^a^	*	NS	*
Liver, g/100 g BW	1.987 ± 0.16	1.854 ± 0.10	2.118 ± 0.15	1.947 ± 0.23	1.959 ± 0.15	1.896 ± 0.04	NS	NS	NS
Abdominal fat, g/100 g BW	1.11 ± 0.11	0.688 ± 0.14	0.622 ± 0.07	0.711 ± 0.07	0.652 ± 0.05	0.699 ± 0.04	NS	NS	NS

Enz: enzyme; CSM: corn silk meal. Values are expressed as the means ± standard error. Means within a row not sharing a common superscript significantly differ from each other. NS, not significant (*p* > 0.05); * *p* < 0.05; ** *p* < 0.01.

**Table 6:** Effects of feeding corn silk meal and/or NSP enzyme on serum biochemical parameters.

	Treatments						ANOVA		
	Control	Enz	4% CSM	8% CSM	4% CSM + Enz	8% CSM + Enz	Enz	CSM	Enz × CSM
Total protein, mg/dL	3.35 ± 0.45	3.45 ± 0.15	3.575 ± 0.14	3.65 ± 0.58	3.9 ± 0.42	3.8 ± 0.18	NS	NS	NS
Albumin, mg/dL	2.175 ± 0.048	2.225 ± 0.18	2.175 ± 0.149	2.213 ± 0.131	2.1 ± 0.158	2.525 ± 0.111	NS	NS	NS
Total cholesterol, mg/dL	140.0 ± 6.4 ^ab^	149.3 ± 5.9 ^a^	130.0 ± 8.5 ^bc^	125.3 ± 5.5 ^bc^	119.8 ± 2.4 ^c^	123.5 ± 2.2 ^bc^	NS	*	*
HDL, mg/dL	30.8 ± 3.3 ^b^	41.84 ± 1.9 ^a^	37.86 ± 2.61 ^ab^	37.51 ± 1.46 ^ab^	39.92 ± 3.32 ^a^	42.75 ± 3.4 ^a^	*	*	*
LDL, mg/dL	90.7 ± 3.5	86.16 ± 7.2	88.44 ± 7.4	85.05 ± 5.1	73.84 ± 7.8	73.85 ± 5.8	NS	NS	NS
ALT, (U/L)	23.75 ± 6.04	24.0 ± 3.24	20.5 ± 2.39	23.0 ± 3.34	21.25 ± 3.30	22.5 ± 5.86	NS	NS	NS
AST, (U/L)	242.3 ± 16.3	234 ± 29.2	244.3 ± 15.9	245.5 ± 14.7	229.8 ± 8.5	226 ± 12.8	NS	NS	NS
Glucose, mg/dL	170.5 ± 16.9 ^a^	144.5 ± 21.56 ^ab^	174 ± 7.9 ^a^	159.8 ± 9.3 ^ab^	121.8 ± 4.7 ^b^	133.5 ± 7.2 ^ab^	NS	NS	*
T3, mg/dL	0.835 ± 0.198 ^b^	1.495 ± 0.154 ^a^	1.358 ± 0.206 ^a^	1.45 ± 0.048 ^a^	1.223 ± 0.186 ^ab^	1.253 ± 0.104 ^ab^	*	*	NS
Titers for ND	1.5 ± 0.29 ^b^	2.25 ± 0.25 ^ab^	3.0 ± 0.41 ^a^	3.25 ± 0.47 ^a^	2.75 ± 0.62 ^ab^	3.5 ± 0.29 ^a^	*	*	*
Titers for H9N1	1.5 ± 0.28	1.5 ± 0.28	1.75 ± 0.25	2.0 ± 0.41	2.5 ± 0.29	2.25 ± 0.47	NS	NS	NS

Enz: enzyme; CSM: corn silk meal; HDL: high-density lipoprotein; LDL: low-density lipoprotein; ALT: alanine aminotransferase; AST: aspartate aminotransferase, T3: triiodothyronine. Values are expressed as the means ± standard error. Means within a row not sharing a common superscript significantly differ from each other. NS, not significant (*p* > 0.05); * *p* < 0.05.

## Data Availability

Data are available up on request.
